# Persistence of Morning Anticipation Behavior and High Amplitude Morning Startle Response Following Functional Loss of Small Ventral Lateral Neurons in Drosophila

**DOI:** 10.1371/journal.pone.0011628

**Published:** 2010-07-16

**Authors:** Vasu Sheeba, Keri J. Fogle, Todd C. Holmes

**Affiliations:** Department of Physiology and Biophysics, University of California Irvine, Irvine, California, United States of America; The Research Center of Neurobiology-Neurophysiology of Marseille, France

## Abstract

Light-activated large ventral lateral clock neurons (large LNv) modulate behavioral arousal and sleep in *Drosophila* while their counterparts, the small LNv (s-LNv) are important for circadian behavior. Recently, it has been proposed that the pattern of day-night locomotor behavioral activity is mediated by two anatomically distinct oscillators composed of a morning oscillator in the small LNv and an evening oscillator in the lateral dorsal neurons and an undefined number of dorsal pacemaker neurons. This contrasts with a circuit described by network models which are not as anatomically constrained. By selectively ablating the small LNv while sparing the large LNv, we tested the relative importance of the small and large LNv for regulating morning behavior of animals living in standard light/dark cycles. Behavioral anticipation of the onset of morning and the high amplitude morning startle response which coincides with light onset are preserved in small LNv functionally-ablated animals. However, the amplitude of the morning behavioral peak is severely attenuated in these animals during the transition from regular light/dark cycles to constant darkness, providing further support that small LNv are necessary for circadian behavior. The large LNv, in combination with the network of other circadian neurons, in the absence of functional small LNv are sufficient for the morning anticipation and the high amplitude light-activated morning startle response.

## Introduction

Pittendrigh and Daan proposed that circadian clocks simultaneously “measure” daily and seasonal changes in day lengths using mutually coupled binary oscillators: the ”Morning” (“M”) oscillator that tracks dawn and the ”Evening” (“E”) oscillator that tracks dusk [1]. While the binary oscillator model was originally developed to explain the peculiar phenomenon of “splitting” and “re-fusion” of morning and evening activity bouts in mammals in response to constant light, this model has recently been adapted to account for the bimodal activity pattern in *Drosophila melanogaster.* Using several genetic and behavioral approaches, attempts have been made to identify the putative “M” and “E” oscillators in circadian neuronal circuit. The “M” and “E” oscillator model is particularly attractive for *Drosophila* as this insect exhibits two distinct bouts of locomotor behavior under 12∶12 h LD cycles - at dawn (morning peak) and dusk (evening peak). Helfrich-Förster [2] suggested that the morning peak in activity is governed by a *per*-independent clock and is entrained by light signals via photoreceptors, while the evening peak is regulated by the circadian clock involving *per* and entrained by CRY (see also [3]). Subsequently, several papers indicate that the “M” and “E” oscillators in *Drosophila* may have distinct anatomical locations as shown by the effects of eliminating different subgroups of clock neurons or by restoring clock gene expression in specific neurons in clock mutants [4]–[7]. These studies conclude that the small LN_v_ function as the “M” oscillator in the *Drosophila* circadian pacemaker circuit, while the LN_d_ and an unspecified number of dorsal neurons function as the “E” oscillator.

PER-null flies lack both morning and evening anticipatory behavior. In an attempt to localize oscillator function between subsets of the LNvs, PER expression was directed in PER-null flies comparing the Mai179 and c929 driver lines. The Mai179 driver line putatively targets the small LNv, while the c929 driver line directs expression to the large LNv along with a large number of non-clock peptidergic neurons, but not the small LNv. Similar claims of small LNv specificity have been made also for the R6 driver line [8]. However, other reports state that the Mai179 and R6 lines drive expression in the both the small LNv and a subset of large LNv [9]. In spite of lacking a specific GAL4 driver for isolating the small LNv for PER expression rescue, the small LNv have been designated as the “morning” neurons. This interpretation of the small LNv as the locus of the morning oscillator appears to have gained some acceptance [6], [10].

In spite of the claim that the M and E oscillators are distinct but coupled [5]–[7] this appealingly simple model must be considered in light of numerous previous studies that show a clear functional contribution by the so-called morning cells (LN_v_) to the evening bout of activity [11]–[13]. In flies lacking functional LNv or their output signal PDF, morning anticipatory activity is absent, and the phase of evening anticipation is advanced [11]–[13]. Ectopic PDF alters oscillator phase [14], [15]. In attempts to reconcile these findings, it has been suggested that the “M” cells modulate the activity of the “E” cells [6], [10]. However, additional work under different genetic and environmental conditions (constant light or constant darkness or alternating low light/darkness) on the *Drosophila* circadian circuit, along with comparison with mammalian circadian circuits, suggests a more complex model of the distribution and coordination of multiple oscillators in *Drosophila* beyond the simple two oscillator model [3], [16]–[22].

We proposed recently that the circadian neuronal circuitry underlying the generation of morning and evening activity peaks is plastic rather than being composed of anatomically fixed oscillators to particular cell types. We posited that oscillator localization varies according to environmental conditions and the overall state of the circadian network (reviewed in [23]; see also [24] and [25]. Furthermore, the relative contribution of the small and large LNv as “morning” neurons is unclear. And while it has been recently demonstrated that non-PDF lateral neurons are modulated by the PDF+ large LNv [8], the question of whether non-PDF circadian neurons in the circuit can interact with the large LNv in a network fashion in the absence of the small LNv to modulate morning behavior remains unanswered. Also, in contrast to anatomically restricted dual oscillator models, large scale imaging and physiological studies of the SCN show that individual oscillators are organized in complex networks [26]–[28].

Electrophysiological analysis indicates that the large LNv exhibit preferential spontaneous firing in both circadian and actual morning [29], [30] and that action potential firing rate of the large LNv is acutely sensitive to light [30]. Recent work further parses the LNv subsets, showing that the large LNv act as light-activated arousal neurons that modulate the circadian circuit for both the morning behavioral peak [30]–[32] (see also [33] and [34]) and the evening behavioral peak [8]. To clarify whether the small LNv are required for the morning behavioral anticipation and peak activity, and to distinguish between acute light versus circadian effects on the light-activated high amplitude morning startle response, we examined these questions using a method developed recently in our laboratory which functionally ablates the small LNv while the large LNv and the other circadian neurons remain functionally intact [31].

## Results

### Peak spontaneous action potential firing rate of large LNv tends to be highest in the morning

**Figure 1:**
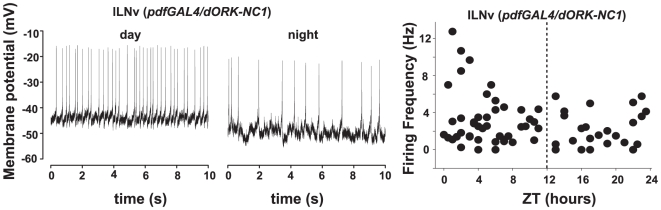
Large LNv highest action potential firing rates occur in the morning. (left, center panels) Representative traces of whole-cell current-clamp recordings of control large LNv (*pdfGal4/dORK-NC1*) taken during the day (left panel, ZT 1) and night (middle panel, ZT 22). Firing frequency (Hz) of 67 individual large LNv neurons plotted according to the time the recording was made, to the nearest half hour from whole brain preparations of flies aged 1–7 days (right panel). Recordings during the day displayed an overall higher firing frequency (3.2±0.4 Hz, n = 43, ZT 0–12) than those taken at night (2.4±0.4 Hz, n = 25, ZT 13–24), the dashed vertical line indicates the time of light-dark transition. The highest large LNv average firing frequencies are observed in the morning (4.5±1.5 Hz, n = 10, ZT 0–2) while the lowest large LNv average firing frequencies tend to occur at night.

Spontaneous action potential firing of large LNv *Drosophila* pacemaker neurons can be measured by whole cell patch in current clamp mode [17], [29]–[31], [35], a recording method adapted from techniques devised for recording from olfactory neurons in adult *Drosophila* whole brain [36]. Flies expressing a membrane delimited GFP marker in the LNv (*pdfGAL4/dORK-NC1-GFP*; [13]) were maintained in standard 12 h∶12 h light:dark cycles. Expression of *dORK-*NC1 in the LNv has no effect either on behavior [13] or membrane electrophysiological properties [30], [35]. Individual flies (1–7 days old) were collected at time points throughout the 24 hr light: dark cycle and whole brains were dissected for whole cell patch clamp recording as described previously in detail [30], [36]. Representative spontaneous action potential firing records are depicted for large LNv recorded under equal illumination conditions at all time points (7 klux, which corresponds to daylight illumination) in the early morning (ZT1) and late night (ZT22) (**Figure 1** **, left and centre panel**). Large LNv spontaneous action potential firing rate recorded under 7 klux light in 67 brains prepared at different phases throughout the entire 24 hour light:dark cycle show that large LNv spontaneous firing can occur throughout the entire light:dark cycle, but that higher firing tends to occur in the morning (**Figure 1, right panel**). Whole cell current clamp recordings of large LNv verify that spontaneous action potential firing peaks in the morning with firing rate gradually decreasing between ZT 0–12 (slope  = −0.19) and increasing between ZT 13–24 (slope = 0.07, **Figure 1, right panel**). Although with this sample size of 67, which is approximately half of the sample size used in [29], they do not reach statistical significance. These results are in qualitative agreement with previous studies that show morning peak large LNv circadian-regulated firing [30] and large LNv peak firing in the morning under diurnal conditions [29], Again qualitatively similar to the results reported in [29], we tend to see more depolarized resting membrane potential values in the morning, however this is not statistically significant by regression analysis (data not shown), while this is reported as significant in [29]. This is probably due to sample size and the variance in large LNv firing rate and resting membrane potential as seen here and in [29].

### Loss of rhythmic circadian behavior in flies expressing neurotoxic Huntingtin-Q128 in the LNv pacemaker neurons

**Figure 2:**
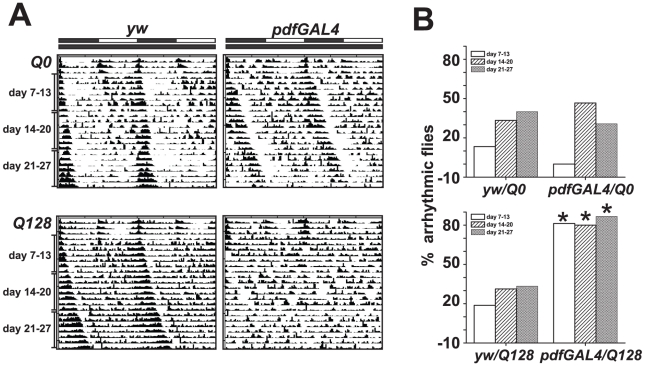
Expression of Q128-Htt in LNv causes loss of circadian locomotor behavior. (**A**) Representative activity/rest records (actograms) of individual male flies expressing either control Q0-Htt (upper panels) or Q128-Htt (lower panels) in the LNv circadian pacemaker neurons. Left panels (*yw*) are genetic background controls where the UAS- PolyQ is not driven by GAL4, while right panels show flies where the expression of Q0-Htt or Q128-Htt is driven by the *pdf*-GAL4 driver in the large and small LNv in the adult brain. Flies were entrained in 12∶12 h light/dark (LD12∶12) regime from age day 2 to 6 following which they were subjected to constant darkness (DD) starting at day 7. The black and white horizontal bars above the top panels indicate the times during which the lights were OFF or ON, respectively, during the initial five days in LD, while the lower black bar indicates darkness during days 7–27. The x-axis indicates the time of day while y-axis indicates consecutive days. Genetic background controls of both Q0-Htt and Q128-Htt lines are predominantly rhythmic throughout the assay in DD. While *pdf* GAL4 driven Q0-Htt expressing flies are not significantly different from their genetic background controls, nearly all Q128-Htt flies are arrhythmic in DD. (B) Percentage of flies with arrhythmic activity/rest pattern during DD day 7–13, day 14–20 and day 21–27 for *pdf*GAL4 driven PolyQ expressing flies (right bars) and their genetic controls (left bars) There is no significant difference in fraction of arrhythmic flies from controls when Q0-Htt is expressed in LNv (upper panel), whereas nearly all Q128-Htt expressing flies are behaviorally arrhythmic for all age groups tested (lower panel). * indicates significant differences at p<0.01; Fisher's exact p test.

Expression of fragments of human Huntingtin (Htt) protein with poly-glutamine (poly-Q) sequences containing greater than 35 copies of Q results in functional neuronal loss and degeneration [31], [37]–[40]. We expressed pathogenic UAS-Q128-Htt (henceforth Q128-Htt) or control non-pathogenic UAS-Q0-Htt (henceforth Q0-Htt) in the LNv using the *pdfGAL4* (*pdf*) driver line and examined locomotor behavior of newly eclosed flies in standard light:dark (LD) conditions for 6 days followed by constant darkness (DD) starting at 7 days of age. For control comparison, the offspring of Q128-Htt and Q0-Htt crossed with *yw* and *pdfGAL4* driver crossed with *yw* were assayed in parallel to test for potential genetic background effects of the UAS lines and the driver. In standard 12 h∶12 h LD conditions, both *pdf/Q0-Htt* and *pdf/Q128-Htt* flies exhibit the typical profile of locomotor activity, with peak activity in the morning and evening along with low levels during mid-day and at night. Similar behavior is observed for both Q0-Htt and Q128-Htt UAS genetic background controls, except that *pdf/Q128-Htt* flies exhibit significantly greater locomotor activity at night at all ages tested (**Supplementary Figure S1, S2**). In contrast, while most control *pdf/Q0-Htt* and genetic background control flies exhibit robust circadian locomotor behavior in constant darkness, the locomotor behavior of most *pdf/Q128-Htt* flies is arrhythmic (**Figure 2A,B**) (** indicates significant differences at p<0.01.) The predominance of circadian arrhythmic behavior of *pdf/Q128-Htt* flies relative to control *pdf/Q0-Htt* flies is stable and seen for days 7–13, 14–20, and 21–27 in constant darkness. Thus, *pdf/Q128-Htt* flies do not later recover from the behavioral circadian arrhythmicity that occurs immediately when exposed to constant darkness. As the small LNv are required for circadian rhythmicity in constant darkness [41], [42], the results indicate that all small LNv neurons are functionally impaired by Q128-Htt expression in young adult flies and that the small LNv contribute to restful behavior at night (**Supplementary Figure S1**).

### Q128-Htt expression in both LNv subsets causes the selective loss of PDF immunoreactivity in the small LNv, but not in the large LNv

**Figure 3:**
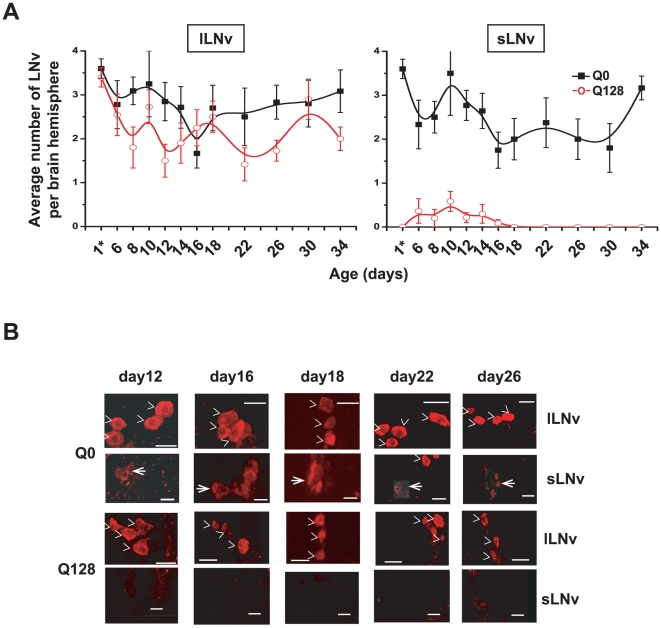
Q128-Htt expression in both LNv subsets causes the selective loss of PDF immunoreactivity in the small LNv. (**A**) The average number of PDF-immunoreactive (PDF+) LNv (± SEM) that can be detected in flies expressing Q0-Htt (black filled square) and Q128-Htt (red hollow circle) in the small and large LNv. The number of PDF+ large LNv remains constant for up to 34 days of age in Q0-Htt and Q128-Htt flies (left panels). The average number of PDF+ small LNv (right panel) remains constant in Q0 lines throughout the assay, while in Q128-Htt flies, PDF+ small LNv are almost never detected between 1–14 days of age, then not detected in any Q128-Htt flies thereafter. (B) Representative confocal maximum projections of anti-PDF staining in large and small LNv in adult flies expressing Q0-Htt or Q128-Htt at ages 12, 16, 18, 22 and 26 days. The PDF+ large LNv (denoted by >) are detectable in all the genotypes up to 26 days of age. The small LNv (thin arrow) are detectable in Q0-Htt flies up to 26 days of age, but in Q128-Htt flies, PDF+ small LNv are rarely detected between 1–18 days of age and never detected after 18 days of age. Scale bars  = 20 µm in large LNv panels and 15 µm in small LNv panels.

Selective vulnerability of specific subsets of neurons is a common feature of neurodegenerative diseases [43]. The small LNv, but not the large LNv, are required for circadian behavioral rhythmicity [41], [42], (see also [23] and [24] for detailed reviews of additional supporting evidence). Thus, the functional loss of circadian rhythmic behavior in *pdf/Q128*-Htt flies suggests that all of the small LNv neurons in the circadian arrhythmic flies are impaired by Q128-Htt expression. To verify this, we measured the levels of the functionally critical circadian neuropeptide pigment dispersing factor (PDF) [12], [13], [17], [44]–[48] levels in both the small and large LNv subsets in flies expressing control Q0-Htt versus pathogenic Q128-Htt driven by *pdf-GAL4* to both LNv neuronal subsets. Anti-PDF immunocytochemical analysis was performed on whole brains dissected from 1–34 day old *pdf/Q0-Htt* and *pdf/Q128-Htt* flies that were maintained under standard LD conditions. There are typically 4–5 large LNv per brain hemisphere and 4–5 small LNv per brain hemisphere in wild type *Drosophila* [49], [50]. Consistent with earlier observations, PDF-positive cell-counts per brain hemisphere in control *pdf/Q0-Htt* flies for both large and small LNv sub-groups ranged between 3–5 cells up to 34 days of age (**Figure 3A**). No consistent loss of PDF-positive large LNv occurs in *pdf/Q0-Htt* or *pdf/Q128-Htt* flies up to 34 days of age, so PDF expression appears to be unaffected by polyQ-Htt expression. In contrast, the numbers of PDF-positive small LNv were severely diminished relative to controls at all ages tested between ages 1–34 days in *pdf/Q128-Htt* flies (**Figure 3A**). No PDF-positive small LNv were observed between the ages of 18–34 days in *pdf/Q128-Htt* flies sampled and they were observed rarely in extremely low numbers in 6–16 day old *pdf/Q128-Htt* flies. Closer examination of the PDF-positive cell bodies shows robust healthy-appearing cells in *Q0-Htt* expressing flies while only small fragments of PDF-positive material is typically seen in the region of the small LNv in Q128-Htt expressing flies at all ages (**Figure 3B**, scale bar is 20 µM for the large LNv panels and 15 µM for the small LNv panels). Under the fixation conditions used, it was not possible to discern whether the small fragments of PDF-positive signal in the region of the small LNv of Q128-Htt expressing flies is cellular or non-cellular. Similarly, membrane delimited GFP expression is selectively lost in small LNv expressing Q128-Htt (*pdfGAL4/dORK-NC1-GFP/Q128-Htt*), but no loss of GFP signal is observed in large LNv expressing Q128-Htt (data not shown).

While loss of PDF-immunoreactivity and GFP expression are clear indications of neural dysfunction, these markers are not equivalent to cell death. To test for polyQ-Htt induced cell death, we performed TUNEL assays using whole mount brains of control and polyQ-Htt expressing flies at various ages. We consistently observed small numbers of TUNEL-positive cells throughout the brain samples for all ages. However, we could detect no specific TUNEL signal in the region of the LNv (data not shown). Thus, there is no direct evidence for polyQ-Htt-induced cell death distinguishable from neuronal dysfunction of small LNv. However, these results indicate that Q128-Htt expression differentially affects the large and small LNv function and suggests that the function of the small LNv are selectively vulnerable to polyQ-Htt protein expression while the large LNv are potentially spared.

### Long-term persistence of spontaneous action potential firing and acute physiological light response in large LNv expressing Q128-Htt

**Figure 4:**
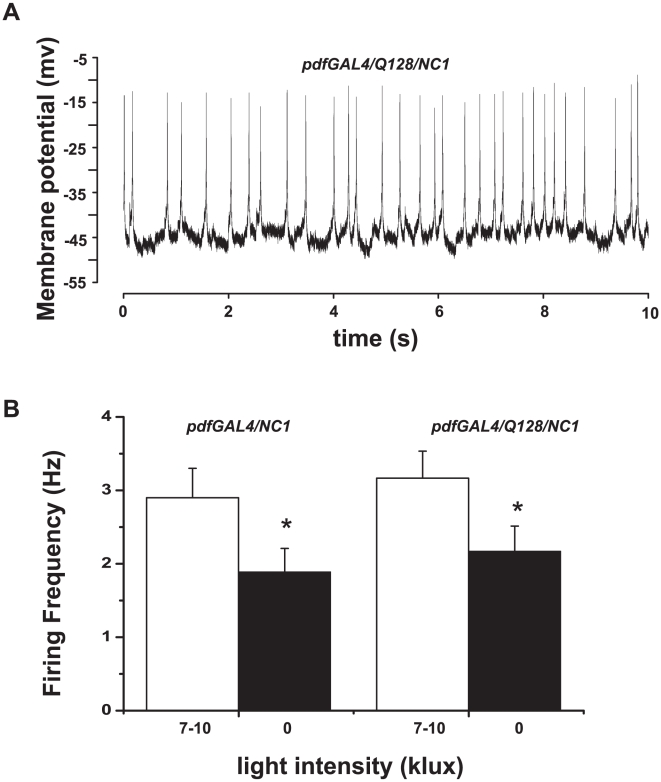
Spontaneous action potential firing and acute physiological light response in large LNv persists in flies expressing Q128-Htt in the LNv. (A) Representative whole cell current clamp recording trace of large LNv in brains of flies expressing Q128-Htt under control of *pdfGAL4* driver shows normal action potential firing in the functional absence of PDF+ small LNv. (B) Upon exposure to 7–10 klux light intensity (white bar) firing frequency is significantly higher (3.2±0.4 Hz) than under darkness (2.2±0.3 Hz black bar; paired *t* test, p<0.001). Controls (*pdfGAL4/NC1*) show similar increased firing frequency under 7–10 klux light intensity of 2.9±0.4 Hz versus 1.9±0.3 Hz under darkness (0 klux). Resting membrane potential is also significantly more depolarized in the presence of light (data not shown). Normal spontaneous action potential firing and light responsiveness persists in large LNv recorded from *pdfGAL4/UAS-Q128-Htt* flies up to 20 days of age.

The results described above suggest that Q128-Htt expression in the PDF-expressing LNv causes selective functional disruption of the small LNv while sparing the large LNv. To test the functionality of the large LNv expressing Q128-Htt, we patch recorded spontaneous action potential firing and physiological light responsiveness of GFP-labelled large LNv from whole brains of 1–20 day old *pdf/Q128-Htt* flies in current clamp mode. Large LNv that express Q128-Htt show normal action potential firing rate and normal firing pattern as shown in recordings made up to 20 days of age. A representative tonic firing neuron is shown in **Figure 4A**, compare this with recording traces of wild type large LNv in **Figure 1** above and in [30], [35]. Large LNv acutely increase their spontaneous action potential firing rate on average by 50% when exposed to daytime levels of light (>2000 lux). This light-induced increase in firing rate is rapid and occurs typically between 1–2 seconds following light exposure and reverses between 1–2 seconds following lights-off [30]. Normal acute large LNv physiological light responses are seen in neurons expressing Q128-Htt up to 20 days of age (n = 12, 11/12 recordings made from flies 7 days old or less; one fly 20 days old), with a significantly higher firing rate during lights-on versus lights-off (**Figure 4B, left panel**). No differences in firing frequency or electrophysiological light response are observed between control *pdfGAL4/NC1* flies and *pdfGAL4/Q128/NC1* flies (**Figure 4B**). There are no apparent age dependent differences in firing rate or physiological light responsiveness in large LNv expressing Q128-Htt (small LNv expressing Q128-Htt cannot be identified for recording as they do not express GFP). From these results and the anti-PDF immunostaining results, we conclude that large LNv are selectively spared while small LNv are functionally impaired following Q128-Htt expression.

### Morning behavioral anticipation and high amplitude morning startle response persist in LD in flies lacking functional small LNv

**Figure 5:**
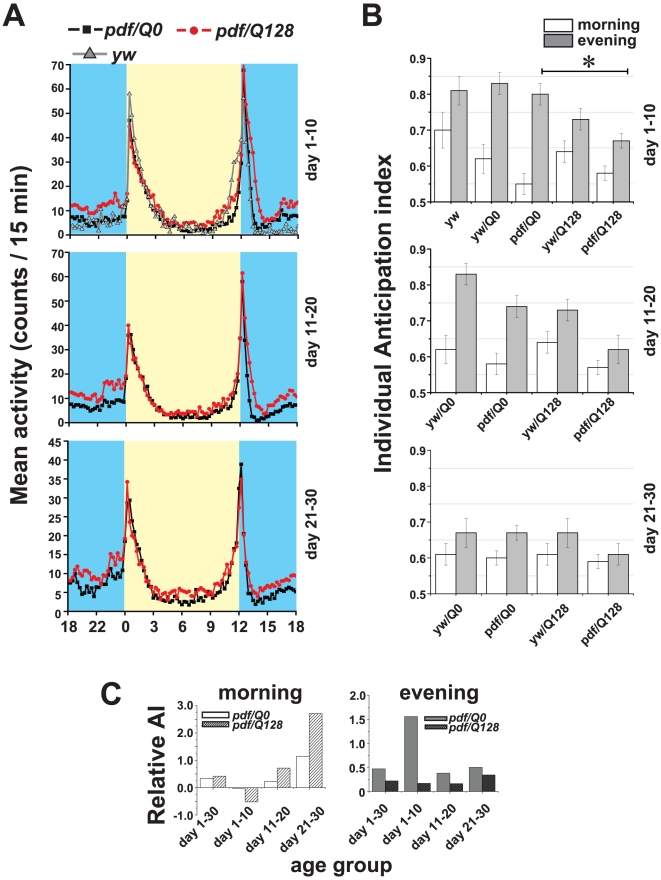
Morning behavioral anticipation and high amplitude morning startle response behavior persists in flies lacking functional small LNv. (A) Mean locomotor activity counts of flies living in standard 12 h∶12 h light:dark cycles with *pdfGAL4* driven expression of control Q0-Htt (black squares), or Q128-Htt (red dots) and yw controls (grey triangles) are shown. Yellow shaded areas denote mean locomotor activity binned in 15 min intervals during the day while blue shaded areas denote mean locomotor activity at night. Average activity is plotted for age day 1–10 (top panel), age day 11–20 (middle panel) and age day 21–30 (lowest panel) to distinguish age dependent effects. (B) Morning and evening anticipation indices were estimated for individual flies at same 3 life-stages as panel A using the fraction of activity during the 3 hours before the transition states of dawn or dusk compared to the activity level through the six hours before transition (Harrisingh et al 2007). One-way ANOVA on the morning anticipation showed no significant differences between the genetic background controls or *yw* controls and the experimental lines *pdf/Q0-Htt* and *pdf/Q128-Htt* flies through all three life stages. Similar ANOVA was performed on the individual evening anticipation indices at all three lifestages. *pdf/Q128-Htt* flies exhibited significantly lower evening anticipation compared to *pdf/Q0-Htt* during the early life stages up to age day 10 (* indicates p<0.01), but during days 21–30 the levels are not different between the two genotypes. But we cannot rule out the non-specific effect of Q128 since *yw/q128-Htt* flies (genetic background controls) did not show significantly higher anticipation than *pdf/Q128-Htt.* (C) Group anticipation indices of morning (left panel) and evening activity (right panel), estimate the positive slope of activity for the 4 hours before dawn ZT0 or dusk ZT12 (Stoleru et al 2004; methods of this paper) for both control *pdf/Q0-Htt* and *pdf/Q128-Htt*. Relative anticipation indices are plotted for both control *pdf/Q0-Htt* and *pdf/Q128-Htt* by normalizing over values of AI for their respective genetic background controls (*yw/Q0-Htt* and *yw/Q128-Htt*). Morning anticipation in flies expressing HTT-Q128 in the LNv appears to be not different from controls up to the age of 20 days and during the advanced age group of 21–30 days they show an enhanced level of morning anticipation suggesting that the network of neurons that regulate anticipatory behavior is plastic and undergoes modifications that allow flies to compensate for the lack of functional sLNv. No statistical comparisons were made as single values for anticipation index is obtained by this method from the average activity profile of each genotype. Similarly evening anticipation also appears unaffected by the loss of functional sLNv (right panel).

Loss of PDF or functional ablation of all PDF-expressing LNv in *Drosophila* leads to the loss of anticipatory behavior preceding the onset of morning, and in some cases that appears to depend on absolute light levels, attenuation of high amplitude light-induced morning startle response [5], [12], [13]. Similar losses of morning (and evening) anticipatory behavior are seen in *per^0^* mutant flies [7]. Directed PER expression to both the small and large subset of LNv in *per^0^* mutant flies rescues the morning peak defect (but not the evening peak defect), while expression directed to small LNv, some of the large LNv, and the majority of the LNd in *per^0^* mutant flies rescues both the morning and evening peak defects. PER expression directed to the large LNv, but not the small LNv, is insufficient to rescue either the morning or evening peak defect under the environmental conditions tested for that study [7]. More recent studies using the Mai179 and R6 driver lines further suggest a specific small LNv contribution to morning anticipation [8]. However, other recent work shows that the Mai179 and R6 driver lines direct expression consistently to a subset of the large LNv as well as the small LNv [9]. To determine the contribution of the small LNv to the morning peak in flies that have functionally intact large LNv and all other circadian neurons, we examined LD behavior in control versus *pdf/Q128-Htt* flies that specifically lack functional PDF-positive small LNv but by all measures retain functional large LNv (**Figures 2** **,** **3** **and** **4**). Visual inspection of averaged locomotor activity of control *pdf/Q0-Htt* and experimental *pdf/Q128-Htt* flies shows similar gradual increases in activity in anticipation of morning and evening; and high amplitude morning and evening startle responses coinciding with lights-on and lights-off in standard 12 h∶12 h LD cycles when examined for 30 days or between days 1–10, days 11–20, or days 21–30 (**Figure 5A**). Similar morning and evening anticipation is seen by visual inspection of averaged behavioral records of control *yw/pdfGAL4*-driver line flies (data not shown).

Visual assessment of gradual increases in activity in anticipation of morning and evening is highly qualitative and imprecise. Several quantitative methods have been developed recently for measuring anticipation index (AI; [5], [51], see Materials and Methods for details). Using the Stoleru method to measure Relative AI (henceforth Group/Relative AI method), control *pdf/Q0-Htt* and *pdf/Q128-Htt* small LNv functionally impaired flies exhibit similar positive values for Relative AI for morning behavior and positive values for evening anticipation over 30 days of 12 h∶12 h LD locomotor behavior (**Figure 5C**). Further analysis using the Relative AI method applied to 10-day successive intervals shows defects in morning anticipation for both control *pdf/Q0-Htt* and experimental *pdf/Q128-Htt* flies between days 1–10, then increasing positive values for Relative AI for morning behavior for days 11–20 and 21–30 for both control *pdf/Q0-Htt* and experimental *pdf/Q128-Htt* flies (**Figure 5C**). The highest Relative AI value is seen for the morning behavior of experimental *pdf/Q128-Htt* flies analyzed between 21–30 days, which is consistent with visual inspection of the average locomotor actograms (**Figure 5A,B**). Relative AI for morning anticipation using this method was also calculated for other control lines, *yw/pdf-GAL4*, and again using this method, defects in morning anticipation can be seen (**Supplementary Figure S1**), AI values not shown. Group/Relative AI values are positive for evening anticipatory behavior for both control *pdf/Q0-Htt* and experimental *pdf/Q128-Htt* flies at all life stages measured with a noteworthy high value for control *pdf/Q0-Htt* flies between days 1–10, again, consistent with visual inspection of average locomotor activity profile actograms (**Figure 5A**). Based on low Group/Relative AI morning anticipation values for some of the time intervals analyzed for two control lines and the lack of robustness of this method for measuring AI, we also analyzed morning and evening anticipation using an AI described in [51] which calculates the amount of locomotor activity in the 3 hours preceding LD transition divided by the locomotor activity during 6 hours preceding LD transition. Unlike the Group/Relative AI, the individual AI method provides a statistical comparison [51]; see also [47]. Using the individual AI method, we calculate positive AI values for both control *pdf/Q0-Htt* and *pdf/Q128-Htt* flies, and see no significant difference in morning anticipation between these two genotypes and their respective genetic background controls (**Figure 5B** **, Figure S1**). The individual evening AI also do not show significant differences between genotypes at any life stage (**Figure 5A,B**; One way ANOVA comparing genetic background controls *pdf/QO* and *pdf/Q128*). Thus, while AI values calculated using either the Relative/Group or Individual methods show a few differences, both analyses yield the same fundamental conclusion that small LNv functionally ablated *pdf/Q128-Htt* flies exhibit measurable morning anticipation similar to controls as well as a high amplitude morning startle response coinciding with lights-on in standard 12 h∶12 h LD cycles when examined for 30 days or between days 1–10, days 11–20, or days 21–30 (**Figure 5A**).

The small LNv functionally ablated *pdf/Q128-Htt* flies do show one interesting consistent difference from *pdf/Q0-Htt* control lines: significantly higher overall night time behavioral activity for all time windows tested (**Supplementary Figure S1B**), indicating that the small LNv may inhibit night time activity, and may act as sleep promoting neurons. Significant increases in overall night time behavioral activity in *pdf/Q128-Htt* flies is robust and has been independently replicated in our laboratory where the genotype of the fly was coded to enable unbiased estimation of activity levels. The *pdf/Q128-Htt* flies with small LNv functionally ablated also show a small but significant increase in total day time behavioral activity (**Supplementary Figure S1B**) These results are consistent with recently published findings from our group and others [31], [33]. Increased day time activity cannot be attributed to phase advanced evening anticipation as UAS control *yw/UAS-Q128-Htt* flies exhibit similar levels of evening anticipation as *pdf/Q128-Htt* flies (**Figure 5A,B**).

### Morning anticipation is retained in DD but the high amplitude circadian morning peak is selectively lost in flies lacking functional small LNv

**Figure 6:**
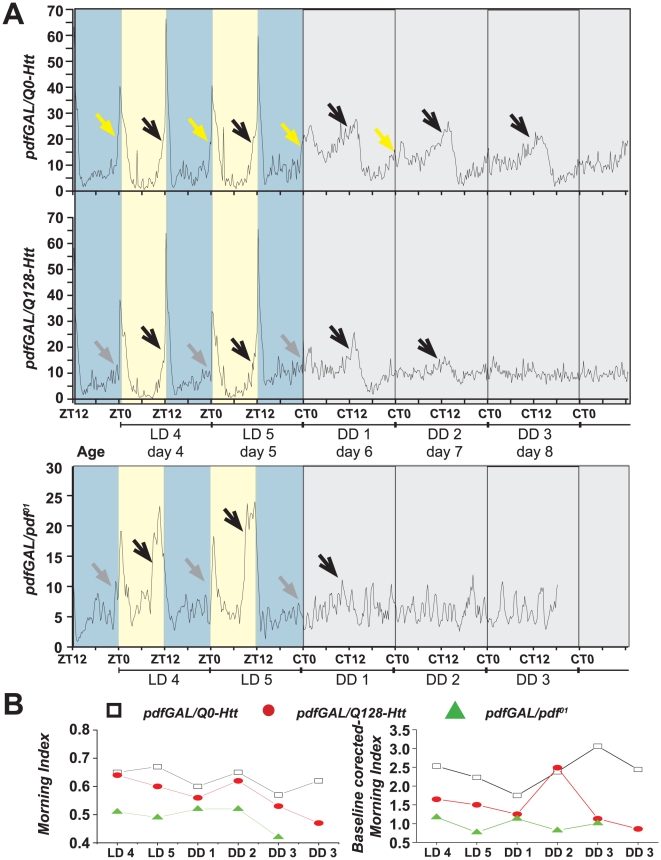
Functional loss of small LNv causes retention of morning anticipation in light-dark cycles, but rapid loss of the circadian-dependent morning peak behavioral activity in constant darkness. (A) Mean activity of flies during the transition from LD12 h∶12 h (day 4 and 5; yellow shaded area represents light phase and blue shaded area represents dark phase) to DD (beginning on day 6; grey shaded area) is shown for control flies (*pdf/Q0-Htt* top panel; averaged across all flies that were rhythmic in DD and smoothed by a moving average of 45 mins - i.e., three consecutive 15 min bins) flies lacking sLNv (*pdf/Q128-Htt* second panel; averaged across all flies that were arrhythmic in DD and smoothed) and *pdfGAL/pdf^01^*controls (bottom panel). Under LD12 h∶12 h both genotypes show robust anticipation of morning (yellow arrow) and evening (black arrow). In control flies the morning peak is clear for at least the first two days in DD along with a persistent robust evening peak. (B) Quantification of morning anticipation activity in LD and first 3 days of DD on data averaged across flies and smoothed across 45 mins. Left panel shows morning anticipation activity index measured as the ratio between activity levels 3 hrs compared to 6 hours preceding lights ON (Harrisingh et al 2007) while right panel shows normalized or baseline-corrected index of morning activity estimated as ratio between the activity levels in the 1 hour preceding lights ON normalized by activity levels at midnight preceding that transition.

Previous imaging and functional studies show that upon introduction to constant darkness molecular oscillations are robust in the small LNv while molecular oscillation quickly dampens in the large LNv; and that the small LNv, but not the large LNv are essential for circadian behavior [5], [7], [11]–[13], [41], [42]. This assertion is also supported by the comparative anatomy of the large and small LNvs [49]. However, circadian molecular oscillations in the large LNv appear to recover after long term exposure to constant darkness (9–14 days), indicating network plasticity [17], [46]. We examined the behavior of control versus small LNv functionally ablated *pdf/Q128-Htt* flies during the transition between standard 12 h∶12 h LD cycles to constant darkness. As seen in earlier experiments under 12 h∶12 h LD conditions, control *pdf/Q0-Htt* and *pdf/Q128-Htt* exhibit both morning anticipatory increase in behavior preceding lights-on and the CT 0 transition from LD to DD (the first “cycle” of darkness). In contrast to control flies, the high amplitude circadian morning peak behavior is entirely absent in small LNv functionally ablated *pdf/Q128-Htt* flies and is thus similar to negative control *pdf^01^* flies (**Figure 6A** third panel; *pdfGAL*/*pdf^01^*). One robust evening peak around CT 12 on day 1 in DD followed by the next weak evening peak (also around CT12) can be discerned approximately 24 hours later on day 2 DD in *pdf/Q128-Htt* flies, after which time, no discernable circadian behavioral rhythms can be observed (**Figure 6A** second panel). In contrast, control *pdf/Q0-Htt* flies exhibit a robust morning peak around CT 0 for days 1 and 2 in DD, and robust evening peaks around CT 12 for days 1–3 in DD (**Figure 6A**). In an attempt to assign a quantitative value to this behavior we adopted two methods both of which involved averaging of activity across flies assayed for each genotype. In the case of *pdf/Q0-Htt* only those flies that were confirmed to be rhythmic in DD were used for the analysis (n = 25; 78% of total flies). In the case of *pdf/Q128-Htt* only those flies that were confirmed as arrhythmic in DD (n = 29; 81% of total flies) were used for further analysis. For both the above genotypes and for negative control *pdfGAL*/*pdf^01^* flies we obtained a measure of morning activity (henceforth morning index Fig. 6B left panel) by estimating the ratio of activity immediately preceding the transition from lights ON to OFF or vice versa (similar to that of [51], except that this was done on activity averaged across flies for each day, see methods). In order to normalize for differences in baseline activity among the three genotypes we also estimated another morning index (normalized morning index) where the activity immediately preceding the lights ON and OFF transitions is normalized by the level of activity at midnight and midday respectively. Both methods clearly show that sLNv ablated flies exhibit morning anticipatory activity which is higher than that of *pdf^01^* flies.

These results indicate that the behavioral morning peak can be readily dissected into two components: a low amplitude anticipatory component that shows small but steady increase for the several hours before lights-on, and a much larger high amplitude morning startle response component that peaks at lights-on. In the absence of functional small LNv, the high amplitude morning startle response component persists in LD indicating that it can be acutely light driven, but disappears immediately upon transition into DD and is thus clock- and small LNv dependent in the absence of light.

## Discussion

Individual pacemaker neurons are capable of autonomously generating circadian rhythms over a large range of phases [52], [53]. This raises the question as to how multiple pacemaker neurons coordinate their activity to generate a single coherent rhythm in overt behavior. Answering this difficult question is potentially more tractable for Drosophila than for mammals due to the relatively small number of pacemaker neurons in the fly circadian circuit and the available tools for fly genetics [50], [54]. Several models have been proposed to account for the general organization of circadian circuits. A dual oscillator model posits that individual oscillators are anatomically restricted to two functionally distinct groups of neurons that control “morning” versus “evening” behavior. The dual oscillator model can be contrasted with a distributed network model which suggests that many cell-autonomous oscillators are coordinated in a more complex, but ultimately flexible fashion (recent reviews of the experimental evidence supporting these respective models can be found in [23]–[25]). While the results above along with several recently published papers [8], [16], [17] supports the idea that there is considerable circuit plasticity in terms of which cells contribute to diurnal and circadian behavioral morning and evening bouts, there is strong consensus that PDF coordinates the entire fly circadian circuit [6], [8], [12], [13], [16], [17], [23], [31], [44], [46], [47], [55], [56]. The small LNv have received considerable attention as being critical for controlling multiple aspects of diurnal and circadian behavior [41], [42], [57], including control of anticipatory behavior immediately preceding the peak in morning behavior [6], [7]. More recent work indicates that the large LNv function as light-driven arousal neurons that modulate sleep [30], [31], [33], see also [32].

To further clarify the relative contributions of the small and large LNv to regulating behavior in light/dark cycles, we made use of the selective functional ablation of the small LNv subset by expression of the neurotoxic protein Q128-Htt [31]. PolyQ protein expression has been shown previously to cause neurodegeneration and functional loss in photoreceptors and other Drosophila neurons [37], [39], [58]. In contrast to the Q128-Htt-induced loss of function of the small LNv, large LNv appear to be spared following expression of Q128-Htt, as shown both by physiological and morphological assays. Such selective vulnerability of certain neuronal populations, while not well understood, is a hallmark feature of neurodegenerative diseases [59]. While pdfGAL4 drives expression in both the small and large LNv neurons, the PDF-positive small LNv precursor neurons appear much earlier in development relative to the PDF-positive large LNv [50].This developmental difference may account in part for the selective functional vulnerability of small LNv to Q128-Htt. PolyQ protein expression in circadian neurons has also been shown to cause abnormalities in courtship behavior [60].

In contrast to the loss of morning anticipation behavior in *pdf^01^* flies, *pdf/Q128-Htt* flies lacking functional small LNv retain morning anticipation along with the high amplitude morning startle response that occurs at the onset of morning at ZT0. Previous work shows that directed expression of PER to the small LNv in a *per^0^* genetic background is sufficient to drive morning anticipation activity [7]. But this interpretation is complicated by the fact that the *Mai179* driver line used for driving expression in the small LNv also drives expression in the three out of six LNd pairs and a smaller subset of the large LNv [7], [20], [61]. Furthermore, the interpretation that PER cycling in the small LNv is sufficient to rescue morning anticipation in a *per^0^* genetic background relies on a rather complicated subtractive analysis: the comparison of PER expression rescue in both PDF-positive LNv subsets for which only morning anticipation (but not evening) is rescued versus lack of rescue of morning or evening anticipation behavior in flies with PER expression directed to the large LNv (and a large number of non-clock peptidergic neurons). The present results indicated that in the absence of small LNv, the morning anticipation behavior persists. This shows clearly that the large LNv operating within the circadian network along with the LNd and dorsal pacemaker neurons are capable of mediating morning anticipation behavior. This is consistent with earlier findings which show that flies retain both morning and evening anticipatory behavior when all their neurons express PER except per-null PDF+ LNv (please refer to Figure 4C of [5]). Taken altogether with all published results, the present results indicate that anticipation behaviors may not be strictly anatomically localized, but rather occur likely as a result of network properties of the entire circadian circuit. Specifically, different combinations of lateral and dorsal circadian neurons are capable of organizing behavior in LD cycles. This conclusion is consistent with [62], who show robust morning and evening anticipatory behavior under different environmental conditions in *per^01^* flies that have PER rescue only in small numbers of DN1 dorsal neurons. Further work shows the DN1 neurons as sensitive output neurons for the circadian circuit [63]. Detailed reviews on localized versus network models of circadian circuit organization can be found in [23], [24].

Close examination of the slope and amplitude of morning anticipation behavior in *pdf/Q128-Htt* flies suggests that this behavior is not as robust as seen in genetically matched controls. This indicates that both the large and small LNv likely contribute to morning anticipation behavior. We employed several published AI methods to quantify morning and evening anticipatory locomotor behavior [5], [51], [55]. While the comparative results showed some differences in the details, we found that using both methods that we could measure morning anticipatory behavior of small LNv functionally ablated flies in standard LD conditions. Using the Relative AI method, we found several examples of control genotype flies that exhibited unexpected apparent defects in morning anticipatory behavior that was inconsistent with visual inspection of averaged locomotor actogram records that suggested intact morning anticipatory behavior. Further examination of the Relative AI reveals that the method is highly sensitive to single transient decreases in averaged locomotor behavior in successive bins prior to lights-on. Due to the formula used by the Relative AI algorithm, this circumstance observed commonly even in control flies leads to negative AI values. Furthermore, the Relative AI method does not provide a statistical comparison between control and experimental groups [5]. These difficulties are overcome by the Individual AI method described in [51].

The large and small LNv appear to functionally interact to produce the high amplitude morning startle response that occurs at the onset of light. Recently the large LNv have been shown by direct patch clamp recording to be acutely light-responsive [30], [31]. Subsequent behavioral and physiological analysis reveals that the large LNv are light activated arousal neurons that promote wakeful behavior [31]. Several other recently published studies suggest that both LNv sub-groups promote wakeful behavior and that the large LNv act upstream of the small LNv [33]; see also [32]. However, we have demonstrated that the large LNv promote wakeful behavior in the absence of functional small LNv and that the small LNv play a minor role in promoting wakeful behavior [31]. Collectively, the results herein and published observations on the large LNv suggest that these cells contribute to the light-driven high amplitude morning startle response that occurs at the onset of light in addition to contributing to morning anticipation behavior. In the absence of functional small LNv and light onset, the high amplitude morning startle response, but not morning anticipatory behavior, is immediately lost in constant darkness. The loss of the small LNv-dependent morning peak is in agreement with previous studies [41]. We conclude that the small and large LNv have both similar and dissociable functions regulating circadian and arousal behavior and that the *Drosophila* circadian circuit operating as a network in the absence of the small LNv is capable of mediating morning anticipation behavior.

## Materials and Methods

### Transgenic Flies and Genetic Crosses

Transgenic Drosophila carrying the 548 amino acids of the human Htt gene downstream of UAS (Upstream Activation Sequence) sites with either a pathogenic polyQ tract of 128 repeats (Htt-Q128) or non-pathogenic form with 0 repeats (Htt-Q0) were obtained from Troy Littleton (MIT, Cambridge, MA). These lines were crossed with LNv specific GAL4- driver line (*pdfGAL4,* [12]).

### Behavioral Assays

Locomotor activity-rest rhythms were assayed by placing individual male flies in glass tubes supplied with food at one end and a sponge stopper at the other end. The movement of the flies across the tube caused breaks in an infra-red beam when these tubes were placed in an automatic Drosophila Activity Monitor (DAM 2, Trikinetics, Waltham, MA). Activity was recorded in 15 min binning intervals. Flies were subjected to light:dark cycles of 12∶12 h for their entire life, or in some cases transferred to constant darkness (DD) Light intensity was between 1000 to 2500 lux during the light phase and below 0 lux during the dark phase and in DD. All assays and rearing of flies were done at 25°C. Raw time series data were obtained from DAM System 3 Data collection software and analyzed using Clocklab software from Actimetrics (Wilmette, IL). Anticipation indices were estimated using two methods – referred to in the text as Stoleru/Relative Index [5] and Harrisingh/Individual Index [51]. To estimate the Stoleru Index, time series data for each life stage (or for all 30 days) of each genotype was averaged across individual flies. Activity counts were binned into 1-hour intervals, then the Relative index was calculated using the formula B_−1_{(B_−1_ – B_−2_) * (B_−2_ – B_−3_)}/B_+1_, where B_i_ corresponds to the activity counts in the *i* ^th^ bin before (−) or after (+) the dark-to-light phase transition for morning anticipation and light-to-dark phase transition for evening anticipation. The Individual Index was calculated for individual flies under LD12∶12 by averaging across either 1–30 days and also for each life stage and then determining the proportion of activity counts during the 3 hours preceding the phase transition over the activity during 6 hours preceding phase transition. Statistical analysis was done using Mann-Whitney U test. For the LD to DD transition assay described in Fig. 6, the Morning and evening indices were estimated using the average across all flies of each genotype per day since day-wise individual fly data had too much variation.

### Electrophysiology

Whole-cell patch clamp recordings were performed during various time points on lLNv of flies maintained under standard LD12∶12 cycles as mentioned in the text as described previously [17]. Briefly, whole brains were dissected and GFP positive large LNv were identified based on their fluorescence using a BX51 WI microscope (Olympus, Lehigh Valley, PA). 2–3 G Ω seals were made in cell-attached configuration following which negative pressure was applied to break into whole cell configuration. Recordings reflect a single recording per brain. Current clamp recordings were made using an Axopatch 200B amplifier (Molecular Devices, Palo Alto, CA), digitized using Digidata 1322A acquisition system (Molecular Devices). Pulse protocols were controlled by pClamp 8.2 Clampex software (Molecular Devices). Data analysis was performed by pClamp 8.2 Clampex software (Molecular Devices). Traces were low-pass filtered by the 3-point Boxcar method and electrical interference filtered at 60 Hz. P/N method was used for leak subtraction.

### Immunohistochemistry and Confocal Imaging

Method for immunohistochemical procedures were as described previously [30]. Briefly, flies of each genotype were sampled within a 3 hour window at age day 1 (under LD 12∶12) and every alternate day starting from day 6 to day 34 (under DD). Around 8–10 males were dissected in ice-cold PBS, treated with collagenase, fixed with 4% paraformaldehyde at room temperature, rinsed and washed with PBS containing 1% Triton-X-100. Next, blocking solution of 10% horse serum in 1% PBS-Triton-X-100 was applied for 30 min at room temperature. The following primary antibodies were applied and incubated overnight at 4°C –anti-PDF (1∶20,000, rabbit, gift from Michael Nitabach, Yale University); anti-Htt (MAb2166, Chemicon,1∶500); anti-elav (1∶100, rat, Developmental Studies Hybridoma Bank). Secondary antibodies used are Alexa 488 (anti-rat), Alexa 555 (anti-mouse), Alexa 647 (anti-rabbit), Alexa 633 (anti-rabbit) from Molecular Probes (Invitrogen, Carlsbad, CA). Brains were mounted on slides using 50% glycerol or FluoroGaurd™ anti-fade reagent (Bio-Rad Laboratories, Hercules, CA) with the ventral side facing upward. Images were obtained using Leica TCS SP2 and Olympus Fluoview1000 confocal microscopes.

### Statistical Analysis

All statistical analyses were performed using Statistica™.

## Supporting Information

Figure S1 (1.91 MB EPS)

Figure S2 (2.52 MB EPS)
